# Preserved learning of implicit regularities with predictive social cues in older adults

**DOI:** 10.3389/fpsyt.2024.1470649

**Published:** 2024-12-10

**Authors:** Claudia Salera, Ala Yankouskaya, Maria Daniela Gazzaneo, Alessia Bonavita, Mariella Pazzaglia, Cecilia Guariglia, Anna Pecchinenda

**Affiliations:** ^1^ Department of Psychology, Sapienza University of Rome, Rome, Italy; ^2^ Department of Psychology, Bournemouth University, Poole, United Kingdom; ^3^ IRCCS Santa Lucia, Rome, Italy

**Keywords:** aging, implicit regularities, learning, attention, gaze cues, attentional bias, joint attention, loneliness

## Abstract

**Introduction:**

With ageing there are changes in the ability to orient attention, which affect more endogenous than exogenous orienting. However, orienting attention by the gaze direction of others shares characteristics of both exogenous and endogenous attention and it is unclear how it is affected by ageing. Being able to orient attention by the gaze direction of others is important to establish successful social interactions (i.e., joint attention), and when gaze direction predicts where in the environment salient events occur, it helps to successfully navigate the environment.

**Methods:**

Here we investigated whether older individuals learn implicit regularities between the direction of eye gaze and a spatial location where salient events occur. We also assessed the association between orienting attention by the gaze of others and loneliness. Seventy older individuals completed the three-items UCLA loneliness scale and the gaze cueing task, in which gaze cues were non-predictive of target location (block 1), but unbeknownst to participants became predictive of the spatial location where the target appeared (block 2) and then, returned to being non-predictive (block 3).

**Results:**

Findings clearly show that older individuals are less able to orient attention by non-predictive gaze cues, but they successfully learn from environmental regularities implemented with direction of eye gaze. This learning biases attention as it enhances the gaze cueing effect even when the regularities are not present. Importantly, gaze cueing was not affected by self-reported loneliness.

**Discussion:**

These findings point to a training strategy to improve joint attention in older individuals.

## 1 Introduction

The ability to orient attention in our environment underlies many daily activities and we use our previous experience to efficiently locate objects and information that are relevant to our current goals. However, with ageing there are changes in this ability, which may differ depending on the modes of attention (see 1, 2). Similarly, there are age-related changes in the ability to learn the relationships between stimuli present in the environment (3). When applied to social stimuli, the ability to orient attention and learn the relationships between social signals plays an important role in preserving good social functions and in establishing social interactions. This is because, shifting attention based on the gaze direction of other people allows to share the object of attention with others (i.e., joint attention) and to attribute mental states as we understand that people look at what they are interested in (4). Ultimately, this ability is fundamental to maintain social connections with others, although it is not clear to what extent it is associated to loneliness, which stems from feeling dissatisfied by current social relationships (5).

Traditionally, attentional orienting has been described as resulting from exogenous or endogenous mechanisms (6, 7). That is, our attention can be voluntarily allocated based on our goals and expectations (i.e., endogenous orienting) or it can be attracted by the characteristic of a stimulus in a reflexive manner (i.e., exogenous orienting). Endogenous orienting is typically studied using a variant of the Posner paradigm (8), in which a symbolic cue (i.e., a stimulus arbitrarily assigned to signal left or right) is presented in the center of the screen, and the to-be-detected target appears either at the cued (valid condition) or at the uncued (invalid condition) location. When symbolic cues are predictive of target location, findings show faster responses to targets appearing at the validly cued location compared to targets appearing at the invalidly cued location, (e.g., 9). The difference in response time between the valid and invalid conditions is known as ‘cueing effect’. Orienting attention by symbolic cues is slow (i.e., it requires a long SOA to allow interpretation of the cue meaning) and occurs only with predictive cues. In a variant of this paradigm, a peripheral cue (e.g., a change in luminance) is presented left or right of a central fixation (e.g., a plus sign), and the to-be-detected target can appear either at the validly or at the invalidly cued location. When peripheral cues are not predictive of target location (i.e., 50% cue predictive validity), findings show faster reaction times (RTs) on valid trials compared to invalid trials. As cueing effects with peripheral cues occur rapidly, at short SOAs (e.g., 100-250 ms), and with cues that are not predictive of target location (i.e., 50% cue predictive validity), orienting of attention is considered involuntary and exogenous.

To date, there is evidence that endogenous orienting of attention elicited by symbolic, predictive cues is compromised by ageing whereas exogenous orienting by peripheral, non-predictive cues is preserved (for a review see 1). Importantly, this typical distinction in exogenous and endogenous modes of orienting attention is challenged by various phenomena, (e.g., 10) among these are orienting attention based on the gaze direction of others (i.e., gaze cueing) and attention guided by learned regularities (i.e., biased and habitual attention). In fact, gaze cueing (11) is considered to share characteristics of both exogenous and endogenous modes of attention as it is observed with central cues like for endogenous orienting, but it also occurs with non-predictive gaze-cues, at short cue-target delay (SOA _=_ 300 ms), and under some conditions, it even elicits Inhibition of Return, which are all typical of exogenous orienting. Indeed, orienting attention by gaze cues relies on the activity of brain regions involved in endogenous attention as well as on those involved in exogenous attention, processing gaze, and mental state attribution (see 12). Interestingly, that gaze direction can orient attention in a way that resembles exogenous orienting is attributed to the direction of eye-gaze being an overlearned directional stimulus as this is an important source of information in social interactions (13; see also 14). Another phenomenon that challenges the exogenous and endogenous distinction is that individuals can learn from environmental regularities, and this learning can guide attention (15). That is, the history of prioritization (of a stimulus or of a spatial location) can induce a habit formation and create an attentional bias. Importantly, it is unclear how ageing affects orienting of attention by gaze direction, as some studies report preserved (e.g., 2, 16) and others reduced (e.g., 17) gaze cueing effects. In fact, in a recent meta-analysis of available evidence, McKay et al. (18) conclude that there is a clear age-related decline in attention and that older individual have reduced gaze cueing effects both with non-predictive and with predictive gaze cues, but that this reduction is more substantial for predictive gaze cues. This conclusion suggests that older individuals would also be less able to be guided by environmental regularities involving predictive gaze-cues. However, given that attention guided by learned environmental regularities and selection history seems to be different from both endogenous and exogenous modes of attention, it is unclear whether older individuals would show enhanced cueing effects when gaze direction predicts the spatial location where an object appears more frequently. That is, it is unclear whether the ability to learn implicit regularities involving gaze direction can induce an attentional bias in older individuals. In fact, with normal ageing there is a decline in learning abilities linked to the function of brain regions, such as the basal ganglia, involved in learning from regularities, but this seems to apply more to explicit than to implicit learning (see 19). Therefore, the present study fills this gap by investigating how older individuals perform on a task that taps on these two phenomena – attentional orienting based on gaze direction and learning implicit regularities between gaze direction and a location where targets occur more frequently. The acquisition of such an attentional bias with gaze cues represents a boundary between endogenous and exogenous modes of attention and it is important as processing social signals and learning from them is one of the key aspects of Theory of Mind and of being able to maintain healthy social connections.

To this aim, we used a gaze cueing task where in the baseline block, gaze-cues predicted target location on 50% of times to assess the cueing effect with typically non-predictive gaze cues. In the second block, unbeknownst to participants gaze cues were predictive on 75% of times, and more so for one spatial location over the other. Older adults may have reduced gaze cueing effect when gaze direction does not predict target location, but their ability to react to a sudden stimulus onset (i.e., exogenous attention) should be preserved. This basic attentional response, which is also what happens when a target appears at the looked at location, remains functional in older individuals (as shown by 20). The question is whether older adults can learn from experiencing these regularities between the direction of eye gaze and target occurrence at the looked at location and to what extent this experience can guide attention also when in the final block, gaze cues are again non-predictive. This allows us to assess whether the regularities implemented in the previous block had been learned, resulting in a habit formation with an attentional bias for the spatial location with more frequent targets. Finally, as the ability to follow the gaze direction of others and learn from these social signals is important for establishing and maintaining social relationships, and older individuals may be more prone to feel lonely (see 21), which can in turn affects how social stimuli are processed (see 22), in a more exploratory vein, we also assessed the relationship between the ability to acquire an attentional bias based on learned regularities with gaze direction and self-reported loneliness.

## 2 Method

### 2.1 Participants

Seventy participants were screened for general mental deterioration with the Mini-Mental State Examination (23). Accordingly, the data of 2 individuals were excluded from the analyses as their score was below the cut-off of 22 (24). The final sample consisted of 68 (F= 41, M= 27) older individuals, age M = 74.07, SE= .81, range 63-88; MMSE corrected score M= 28.07, SE= .15, range 23-31; years of education, M= 11.5, SE= .51, range 5-18. In addition, participants completed the three-item UCLA loneliness (25, 26).

We did not calculate sample size a priory due to a lack of studies using the same experimental design (27). However, using G*Power, version 3.1.9.6 (28), we computed the sensitivity of our critical analysis to detect the cueing effect across the three phases. Results showed that with our sample of 45 participants, considering α = 0.05 and 1 - β = 0.80, the minimum effect size that could be detected is η^2p^ = 0.03 (Cohen’s f = 0.19) corresponding to a small-medium effect size.

All participants gave their informed consent, which was obtained according to the Declaration of Helsinki (1991). Participants had normal or corrected-to-normal vision and were naïve to the purpose of the study. The study was approved by the institutional ethic committee (approval number 0000867, 28/04/2021).

#### 2.1.1 Stimuli and apparatus

Grayscale photographs (10 by 8 cm) of four actors (2 females, 2 males) with neutral expressions were selected from the Radboud dataset (Faces IDs: Rafd090-12; Rafd090_15; Rafd090-25; Rafd090-26; 29). Based on published validation ratings (29), faces with the most recognizable expressions were selected (e.g., 30). For each actor, one photograph gazing left and one photograph gazing right were selected. Each photograph was converted to grayscale and faces were cropped in ovals. Central fixation was a cross subtending a visual angle of 0.4° x 0.4°. The cue was a face with eyes looking left or right and subtended a visual angle of 9° x 6°. The target was either a “L” or a “T” subtending a visual angle of 1.2° x 0.8° and it appeared 12° to the left or right of the center of the screen (i.e., fixation point). All stimuli were presented on a white background.

Stimulus presentation and data collection were controlled using Testable software (31). Stimuli were presented on a Lenovo laptop (ideapad 3-15IML05 81WB) running Windows 11 with a 15.6-inch monitor (resolution 1920 x 1080, refresh rate 144 Hz). Responses were collected using the built-in keyboard of the portable computer.

### 2.2 Procedure

**Figure 1:**
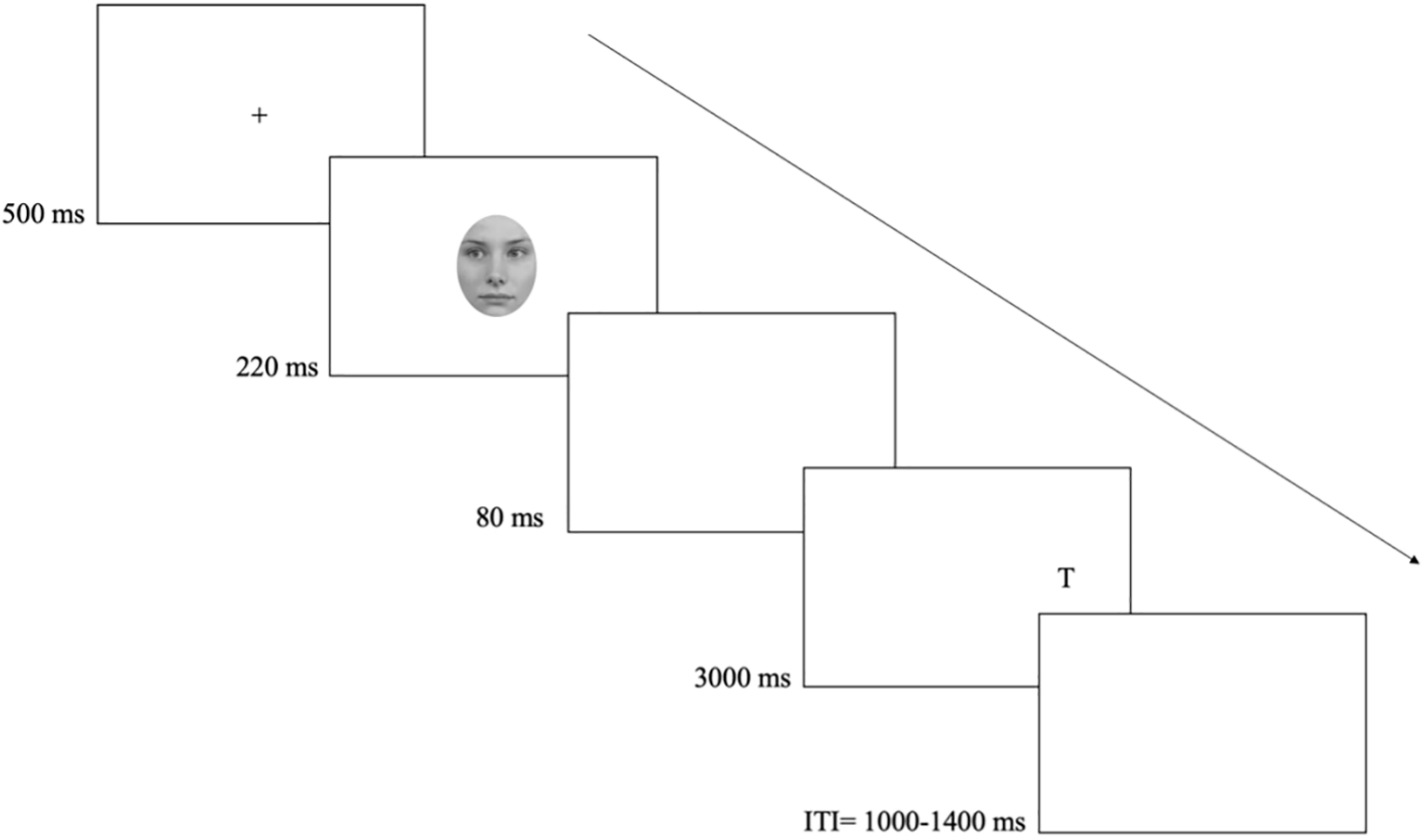
Events and timing of a typical trial of the gaze cueing task. The example shows an invalid trial with the face-cue looking left and a target presented on the right.

Upon completion of the consent form, participants were screened using the Mini-Mental State Examination (MMSE, 23) scale, after which they completed the three-item UCLA loneliness scale (25, 26). Next, participants were invited to sit comfortably in front of a computer screen, at viewing distance of approximately 45 cm (i.e. the distance from the chair to the computer-desk), and in keep with past studies (e.g., 2, 32), without using a chinrest. Participants were instructed to maintain fixation (i.e., to look at the cross) and respond as fast and as accurately as possible to the letter identity (i.e., the target) – L or T – by pressing one of two adjacent keys chosen to be perpendicular to the left and right target position (‘m’ and ‘k’ keys) and accordingly labelled. Key assignment to targets was counterbalanced between participants. Each trial started with the display of the central fixation cross. After 500 ms, the cue appeared for 220 ms (as in 32). After 80 ms (300 ms SOA) the target (L or T) appeared either left or right. The target remained on the screen until a response or 3000 ms had elapsed. To prevent strategies based on temporal expectancies (e.g., 33), the ITI varied randomly between 1000 and 1400 ms in steps of 100 ms (see **Figure 1**).

After 32 practice trials with non-predictive cues (50% cue validity), participants completed a total of 336 trials divided in three blocks of 112 trials each. Between blocks, participants had the opportunity to take a break. In the first (baseline) and last (testing) blocks, cues were non-predictive of target location (50% cue validity), whereas unbeknownst to participants in the second block (learning), the predictive validity was 75% and gaze cues were more predictive of targets occurring at one spatial location (i.e., the location rich with targets). Therefore, in the baseline and testing, half the trials (i.e., 56 trials) had valid cues (of which, 28 trials with target on the left), and the other half had invalid cues (of which 28 trials with target on the left), equally distributed between the two spatial locations. In contrast, in the learning block, there were 84 trials with valid cues and 28 with invalid cue and of the 84 trials with valid cues, the targets occurred more often (i.e., for 64 trials) at one location (designated “rich”) and for the remaining 20 trials, targets occurred at the other location (designated “scarce”), see **Table 1**.

**Table 1:** Schedule of valid and invalid cues for the two spatial locations in the three phases of the task.

Baseline 50% cue predictive validity		Learning 75% cue predictive validity		Testing 50% cue predictive validity	
50% valid left	50% valid right	75% valid left	25% valid right	50% valid left	50% valid right

The example shows “rich location” to the left. Assignment of left or right to the rich location was counterbalanced across participants.

At the end of the task, participants responded to three questions: 1) did you note any relationship between gaze direction and the letter to which you responded? (yes/no); 2) Do you think gaze direction was linked to the position where the letter appeared? (yes/no); 3) Could you guess how often the letter to which you responded appeared at the location looked at by the face? (0= never, 100= always).

### 2.3 Experimental design

The experimental design is a 3 (Phase: baseline, learning, testing), by 2 (Cue: valid, invalid) by 2 (Location: rich, scarce).

### 2.4 Data analyses

For reaction times (RT), values from trials with errors (2.34%) and values below 120 ms (0.04%) or 2.5 SD above the overall mean (5.06%) were not analyzed (7.44% in total). Mean RTs and response accuracy were computed for each experimental condition.

For the three-items UCLA loneliness scale (25, 26), which assesses respectively relational connectedness, social connectedness, and self-perceived isolation, a total score was computed by adding the value of each individual question, M= 4.99, SE= .18, range 3-9, Median= 5. Based on the median value, participants were divided in low (N= 30) and high (N=38) loneliness groups.

Data were analyzed with a 2 (Group: low vs high UCLA loneliness score) by 3 (Phase: baseline, learning, testing) by 2 (Cue: valid, invalid) by 2 (Location: scarce, rich) mixed factorial ANOVA with the first factor between subjects. However, findings showed no statistically significant main effects or interactions involving the Group factor. Therefore, data were analyzed with a 3 (Phase: baseline, learning, testing) by 2 (Cue: valid, invalid) by 2 (Location: scarce, rich) repeated measures ANOVA. All pairwise comparisons are Bonferroni-corrected. Please, note that we use the rich/scarce labels for Location (rather than left and right) to reflect the regularities implemented during the testing phase. Finally, Pearson’s correlations were computed between the gaze cueing index, MMSE scores, the total score as well as the individual items of the three-item UCLA loneliness scale.

## 3 Results

### 3.1 RTs

Results of the Mauchly’s Test showed a significant violation of sphericity for Phase, W = .74, Chi-square (2) = 20.04, p <.001, for Phase by Cue, W = .87, Chi-square (2) = 8.95, p = .011, Phase by Location, W = .81, Chi-square (2) = 13.58, p = .001, and for the 3-way Phase by Cue by Location interaction, W = .90, Chi-square (2) = 7.85, p =.020, therefore the Greenhouse-Geisser corrected p values are reported. ANOVA results showed a significant main effect of Phase, F(2, 134) = 4.59, p = .019, η^2p^ = .064 due to faster RTs during testing (M= 877, SE= 23) than during learning (M= 897, SE= 23), p= .006 but no difference with the baseline (M= 901, SE= 24), n.s. The Cue F(1, 67) = .028, p = .87, and Location F(1, 67) = .87, p = .36 main effects were not statistically significant. The Phase by Cue F(2, 134) = .090, p = .91 and the Phase by Location F(2, 134) = .137, p = .87 interactions were not statistically significant. In contrast, the Cue by Location F(1, 66) = 36.97, p = .001, η^2p^ = .35 and the Phase by Cue by Location interactions were statistically significant, F(1, 66) = 36.97, p = .001, η^2p^ = .35, see **Table 2**.

**Table 2:** Mean RTs (and SEs) for the 3-way interaction, Phase (Baseline, Learning, and Testing) by Cue (Valid and Invalid) by Location (Rich and Scarce).

	Cue	Baseline	Learning	Testing
Rich	Valid	899 (24)	863 (22)	864 (22)
	Invalid	901 (25)	928 (24)	888 (24)
Scarce	Valid	902 (24)	932 (24)	890 (24)
	Invalid	904 (25)	867 (22)	865 (23)

The 2-way interaction was due to faster RTs on trials with valid cues (M= 876, SE= 22) than on trials with invalid cues (M= 906, SE= 24) for the rich location, p<.001, indicating the typical cueing effect. In contrast, the reversed pattern was present for the scarce location as RTs were slower on trials with valid cues (M= 908, SE= 23) than on trials with invalid cues (M= 879, SE= 23), p<.001. The 3-way interaction was analyzed using the cueing index, which was computed for each phase as the relative change in overall response speed according to the formula (e.g., 22, 30, 34–36):


[(RT_Invalid−RT_Valid) / (RT_Invalid+RT_Valid)/2]∗100.

**Figure 2:**
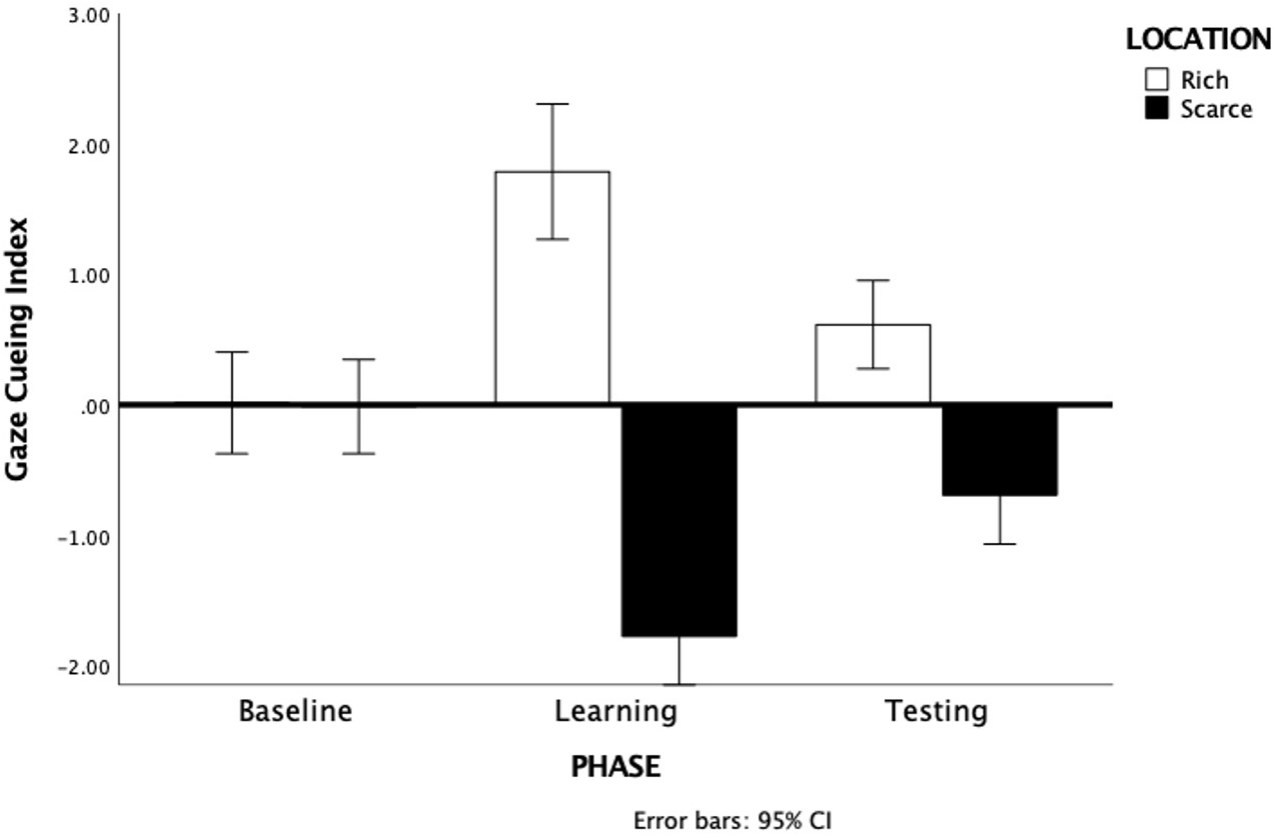
Gaze Cueing Index for the rich and scarce locations during the 3 phases. Note that rich and scarce does not apply to the baseline. Error bars are 95% Confidence Intervals.

Relative difference scores were analyzed with a 3 by 2 repeated-measures ANOVA, with Phase (baseline, learning, testing) and Location (rich, scarce) as within-subject factor. All pairwise comparisons are Bonferroni-corrected. Results showed a significant main effect of Location F(1, 66) = 366, p <.001, η^2p^ = .357 due to a larger cueing index for the rich (M= .81, SE= .16) than for the scarce location (M= -.83, SE= .15) for which the cueing index was negative (i.e., reverse cueing). This was qualified by a significant Phase by Location interaction F(2, 132) = 64.95, p <.001, η^2p^ = .496 (see **Figure 2**).

Pairwise comparisons showed an equally small cueing index in the baseline and no differences between the two locations, p= .93. In contrast, the cueing index in the learning phase was much larger for the rich location (M= 1.79, SE= .26) than for the scarce location (M= -1.75, SE= .19), p<.001. Importantly, the same pattern was present in the testing phase with a larger cueing index at the rich location (M= .61, SE= .17) than at the scarce location (M= -.69, SE= .19), p<.001 indicating that participants had acquired an attentional bias that persisted also after the regularities between gaze-cues and spatial location had been removed.

### 3.2 Response accuracy

Results of the Mauchly’s Test showed a significant violation of sphericity for Phase, W = .67, Chi-square (2) = 26.45, p <.001, for Phase by Cue, W = .61, Chi-square (2) = 32.73, p <.001, Phase by Location, W = .83, Chi-square (2) = 12.25, p = .002, and for the 3-way interaction Phase by Cue by Location, W = .91, Chi-square (2) = 6.44, p = .04, therefore the Greenhouse-Geisser corrected p values are reported. ANOVA results showed only a statistically significant Phase by Cue interaction, F(2, 132) = 3.58, p = .031, η^2p^ = .05. Pairwise comparisons showed that response accuracy on trials with valid and invalid cues did not differ in the baseline, p=.21 and testing, p=.67 but in the learning, it was greater on trials with valid cues (M= .98, SE= .004) than on trials with invalid cues (M= .97, SE= .004), p=.035. No other main effects or interactions were statistically significant.

### 3.3 Post-task questions

Only 8 participants (11.7% of our sample) thought that there was a relationship between gaze direction and the target letter (question 1) and only 3 participants (4.4% of our sample) thought that gaze direction was linked to the position of the target letter. Interestingly, only one participant answered positively to both questions but when asked to guess how often the target letter occurred at the location looked at by the face (question 3) the answer was 50%. Only 9 participants (13.2% of our sample) thought that the target appeared at the cued location more than 50% of times (question 3). This suggests that participants were at least not able to verbally report the regularities that affected their behavioral responses at the cueing task.

### 3.4 Correlations

Pearson’s correlations showed only a significant negative correlation between MMSE score and relational connectedness of the three-item UCLA loneliness scale, r= -.257, p= .035.

## 4 Discussion

The present study investigated whether older individuals orient attention based on the gaze direction of others when gaze direction is not predictive as well as when it is predictive of a salient event (i.e., the target), and whether they learn implicit regularities between the direction of eye gaze and the location where salient event occurs. The research question stems from evidence that there is an age-related decline in orienting attention that is greater for endogenous than for exogenous attention (e.g., 18) but also that orienting attention by the gaze direction of others shares characteristics of both exogenous and endogenous attention and relies on partially segregated neural mechanisms (see 12). In addition, attention can be guided by learned regularities, which can be affected by age-related changes in basal ganglia function, (see 19). However, it is unclear whether older individuals can acquire an attentional habit by learning implicit regularities between the direction of eye-gaze and the spatial location where targets were more frequently presented: the present findings clearly show that they do.

More specifically, we used the gaze cueing task and – based on previous studies with older adults – we used a SOA of 300ms, a cue presentation of 220ms (e.g., 17, 32), and static cues with averted gaze (e.g., 37, 38). Static gaze cues (i.e., presenting a still image with averted gaze) may have limited ecological value compared to dynamic gaze cues, which entail presenting a still image of a face with straight gaze, followed by a still image of the same face with averted gaze, and therefore convey implied motion. However, although in young individuals the cueing effects observed with dynamic gaze cues are typically greater than those observed with static gaze cues (e.g., 22), dynamic gaze cues are not immune to limitations as attention may be shifted mechanically in the direction of another’s gaze. Indeed, it has been argued that orienting attention with static gaze cues may require understanding the mental state of others such that people look at what they are interested (e.g., 39). Moreover, dynamic gaze cues do not only rely on the ability to shift attention based on the direction of eye gaze but also on being able to disengage attention from the face with straight gaze (i.e., looking at the observer), which older adults may have difficulties in doing. Therefore, older individuals may show small, or no gaze cueing effects simply because they have a difficulty disengaging attention from the face with straight gaze.

In the present study with these task parameters, older individuals showed very small gaze cueing effects in the baseline block, which is not unusual (see 2, for a review of the effects of age on gaze cueing see 14). Importantly, the present findings were obtained with the typical task parameters used in gaze cueing tasks with older individuals, including allowing for a more ecological setting where head as well as gaze movements may follow the direction of the social cue (40). In fact, it has also been argued in favor of using videos as more ecological stimuli, and recent evidence points to good size gaze cueing effects in older individuals when using videos of actors shifting their gaze from straight to averted (2). Having used static gaze cues, this finding cannot be due to older individuals’ difficulty in disengaging their attention once established eye contact with the central face.

In our study the gaze cueing effects were much larger in the learning block, when gaze direction was predictive of target occurrence and of where the target would occur. In fact, gaze cueing effects were larger for the rich location where targets were more frequently presented. This aspect represents the strength of the current task manipulations as the biased target distribution entailed that the high target probability for the rich location increases tonic alertness, resulting into an additive effect on the magnitude of spatial orienting by gaze cues (i.e., 41). On the other hand, this same aspect of the task manipulation limits the current findings to learning regularities that entails exogenous events such as target occurrence to a spatial location rather than purely endogenous events such a cue predictive validity. Importantly, this effect did not only occur when the regularities were in place, but it continued also once they were removed in the testing block. Therefore, older individuals detected and learned from the implicit regularities between an exogenous event (i.e., the target) and a spatial location and this learning, biased attention, enhancing the gaze cueing effect. Indeed, there is evidence that implicit associative learning involving non-target information is preserved in older adults (14), which suggests that what declines with age is the ability to learn explicit contingencies rather than implicit regularities (see 19). It should also be mentioned that this biased attention affected mostly response speed once the regularities were removed (i.e., in the testing phase) and it was not due to a speed accuracy trade-off as participants were faster but not less accurate on validly cued trials than on invalidly cued trials.

Finally, it is interesting to note that the present findings were observed for both, older individuals with higher levels of loneliness and older individuals with lower levels of loneliness and that self-reported loneliness did not correlate with gaze cueing index. We had hypothesized that loneliness may motivate more toward social signals, resulting in larger gaze cueing effects. That this was not the case in the present study is in keep with findings from our laboratory, showing that loneliness does not affect gaze cueing effects in young individuals (Pecchinenda, Yankouskaya, Gonzalez-Pizzio, under review). Although caution is in order when interpreting a null effect, the present findings suggest that acquiring an attentional bias based on learned regularities with a social signal is not affected by self-reported loneliness in older individuals. However, our assessment of the role of loneliness on learning associations with social stimuli was exploratory, and future studies may attempt to enhance the difference between high and low loneliness for instance, by pre-selecting individuals with higher and lower levels of loneliness. The only significant, albeit small, negative association was observed between relational connectedness and MMSE scores, such that worse cognitive functions as indexed by a lower MMSE score are associated to higher levels of self-reported relational connectedness. However, this finding is at odds with a growing literature of a link between loneliness and cognitive decline in the elderly (e.g., 22, 42), and future research should look in depth to better understand the possible factors underlying this association.

The present findings have theoretical implications as they suggest that orienting attention by gaze direction and orienting attention by an acquired habit may reflect different subsystems of spatial attention, with the latter being spared by ageing. Importantly, the present findings have also practical implications as they point to a training strategy that may help to contrast age-related decline in joint attention and improve attention orienting based on gaze direction in older individuals.

## Data Availability

The datasets presented in this study can be found in online repositories. The names of the repository/repositories and accession number(s) can be found below: Open Science Framework: https://osf.io/dzhfy/?view_only=23b65206c20b4361986edbfb0ad58654 .
